# Impact of *ex vivo* Sample Handling on DNA Methylation Profiles in Human Cord Blood and Neonatal Dried Blood Spots

**DOI:** 10.3389/fgene.2020.00224

**Published:** 2020-03-24

**Authors:** Aya Sasaki, Bona Kim, Kellie E. Murphy, Stephen G. Matthews

**Affiliations:** ^1^Department of Physiology, University of Toronto, Toronto, ON, Canada; ^2^Lunenfeld-Tanenbaum Research Institute, Sinai Health System, Toronto, ON, Canada; ^3^Department of Obstetrics and Gynaecology, University of Toronto, Toronto, ON, Canada; ^4^Department of Medicine, University of Toronto, Toronto, ON, Canada

**Keywords:** DNA methylation, cord blood, blood cards, *ex vivo* storage, reduced representation bisulfite sequencing

## Abstract

The profiling of DNA methylation modifications in peripheral blood has significant potential to determine risk factors for human disease. Little is known concerning the sensitivity of DNA methylation profiles to *ex vivo* sample handling. Here, we studied typical conditions prior to sample storage associated with cord blood samples obtained from clinical investigations using reduced representation bisulfite sequencing. We examined both whole blood collected shortly after birth and dried blood spots, a potentially important source of neonatal blood for investigation of the DNA methylome and the Developmental Origins of Health and Disease in human cohorts because they are routinely collected during clinical care. Samples were matched across different time conditions, as they were from the same cord blood samples obtained from the same individuals. Maintaining whole blood *ex vivo* up to 24 h (4°C) or dried blood spots up to 7 days (room temp.) had little effect on DNA methylation profiles. Minimal differences were detected between cord blood immediately frozen and dried blood spots. Our results indicate that DNA methylation profiles are resilient to *ex vivo* sample handling conditions prior to storage. These data will help guide future human studies focused toward determination of DNA methylation modifications in whole blood.

## Introduction

Studies of gene expression have demonstrated that *ex vivo* incubation time alters the transcript abundance of many genes (Hartel et al., 2001; Rainen et al., 2002; Baechler et al., 2004; Debey et al., 2004). These genes are involved in the activation of stress and inflammation-induced pathways, cell cycle progression and apoptosis. Thus, the timing and procedures associated with blood collection are critical for accurate and sensitive measurements of the state of gene activity.

Assessment of DNA methylation in peripheral blood has significant potential for determining risk factors for human disease. As genomic-scale arrays and sequencing technologies are increasingly applied to the study of peripheral blood, it is important to consider the variables that may affect the interpretation of data. Storage times and temperatures often vary significantly from one laboratory to another because of differences in collection, transport, and processing of human whole blood. However, little is known concerning the sensitivity of DNA methylation profiles to *ex vivo* sample handling conditions.

Neonatal dried blood spots are a potentially important source of neonatal blood for investigation of the DNA methylome and the Developmental Origins of Health and Disease in human cohorts because they are routinely collected during clinical care. There has also been increasing interest in the use of dried blood spots for epigenome-wide analysis, as DNA methylation profiles extracted from dried blood spots are strongly correlated with those of freshly collected blood (Hardin et al., 2009; Aberg et al., 2013; Hollegaard et al., 2013; Joo et al., 2013; Ghantous et al., 2014).

In the present study, we aimed to determine the effects of *ex vivo* sample handling prior to DNA extraction on DNA methylation profiles in cord blood. Our goal was to simulate common variations in the conditions under which cord blood is maintained prior to storage for research and clinical assessments using the same cord blood samples obtained from the same individuals. We examined whole cord blood collected within 30 min of birth and either immediately frozen or kept at 4°C. We also examined blood dried on Guthrie cards at room temperature to simulate procedures for neonatal blood collection for clinical assessments.

## Materials and Methods

### Subjects and Blood Samples

**FIGURE 1:**
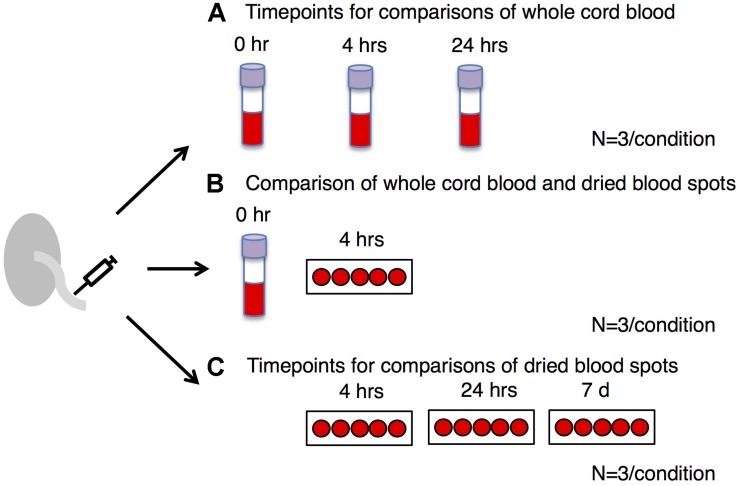
Study overview. We examined DNA methylation profiles in whole blood: **(A)** treated with EDTA and frozen immediately after collection (0 h) compared to whole blood frozen after 4 h or 24 h at 4°C, **(B)** EDTA-treated dried blood spots frozen after 4 h at room temperature compared to whole blood frozen immediately, and **(C)** non-EDTA-treated dried blood spots frozen after 4 h, 24 h or 7 days at room temperature. The same samples obtained from the same individuals (*n* = 3) were used for each time point.

A study overview is presented in Figure 1. Healthy subjects (*n* = 7) were recruited by Mount Sinai Hospital and the Research Centre for Women’s and Infants’ Health BioBank (Toronto, ON, Canada). This study was approved by the Mount Sinai Hospital Research Ethics Board (MSH REB# 17-0210-E) and the University of Toronto Research Ethics Boards. Umbilical cord blood from each subject was collected within 30 min of birth. For 4 subjects (Figures 1A,B), blood was deposited into four EDTA purple top vacutainers (Becton Dickinson). The first EDTA tube was frozen immediately and stored at −80°C. The second and third EDTA tubes were kept at 4°C for 4 and 24 h, respectively, and then stored at −80°C. The fourth EDTA purple top vacutainer was sent to Mount Sinai Services (Mount Sinai Hospital, Toronto, ON, Canada) for complete blood count (CBC) measurement using Sysmex XN-9000 (Sysmex). The purpose of CBC measurement was to reveal the distribution of different types of nucleated cells across subjects and examine if they were within the expected range for neonates. Part of the EDTA-treated cord blood was placed on a Guthrie card (75–80 μL of blood per spot; Whatman 903), left to air-dry for 4 h at room temperature and then stored at −80°C. One subject yielded insufficient blood for CBC or time course analysis. For three subjects (Figure 1C), blood was placed directly onto a Guthrie card without EDTA treatment (75–80 μL of blood per spot; Whatman 903). Cards were left to air-dry for 4 h, 24 h or 7 days at room temperature and then stored at −80°C. All samples were derived from singleton pregnancies delivered at term (≥37 weeks). Patients with gestational diabetes, diabetes, or hypertension were excluded.

### DNA Extraction and Quantification

DNA was extracted from whole blood and the blood spot cards using methods described previously (Ghantous et al., 2014), with minor modifications. Briefly, one half of a single blood spot was cut into small pieces and incubated with lysis buffer (1218 μL) and Proteinase K (33 μL) using Gensolve reagents (GenTegra: GVR-113) in an Incubator/vortex shaker set at 1400 rpm for 90 min, at 56°C (ThermoMixer^®^ C, Eppendorf). The rest of the procedure followed the manufacturer’s protocol. The lysate was processed using a QIAamp DNA Blood Mini Kit (Qiagen: Cat. #51104). The extracted DNA was quantified with a Quant-iT PicoGreen dsDNA assay (ThermoFisher: Cat.# P11496) and DNA quality was assessed by TapeStation (Centre for Applied Genomics in the Hospital for Sick Children, Toronto, ON, Canada). Samples with DNA Integrity Numbers (DINs) over 7 were used for further analysis.

### Reduced Representation Bisulfite Sequencing (RRBS)

Reduced representation bisulfite sequencing (RRBS) libraries were generated from 100 ng of high-quality dsDNA for each sample using the RRBS Methyl-Seq System 1–16 (Ovation: Part # 0353) and EpiTect Fast DNA Bisulfite kit (Qiagen: Cat. #59824) following the manufacturer’s protocols. *Msp*I restriction enzyme digestion and size selection were used to enrich libraries for gene regulatory elements containing CCGG motifs. The resulting libraries showed three peaks on the Bioanalyzer High Sensitivity DNA Chip (i.e., 200, 265, and 330 bp) due to expected *Msp*I-containing micro-satellite repeats in the human genome as well as the absence of peaks indicating unligated adapters. Representative output of the Bioanalyzer High Sensitivity DNA Chip showing three peaks are available in the manufacturer’s protocol (Ovation: Part # 0353). All libraries generated in this study met these criteria prior to sequencing. RRBS libraries meeting this criterion were sequenced in multiplexes of up to 10 samples, balanced by condition (Donnelly Sequencing Centre, University of Toronto, Toronto, ON, Canada) on a NextSeq500 (Illumina) following the manufacturer’s protocols for single end reads at 75 bp read length.

### Differentially Methylated CpG Sites (DMCs)

Adaptor sequences were trimmed using Trim Galore^1^ followed by additional filtering and trimming using a python script provided by NuGEN to remove reads that did not contain an *Msp*I site signature at the 5′ end. The python script is available on GitHub^2^. The reads were then aligned using Bismark (Krueger and Andrews, 2011) and sorted by Samtools (Li et al., 2009). We then used MethPipe/Radmeth (Song et al., 2013; Dolzhenko and Smith, 2014) to identify significant differentially methylated CpG sites (DMCs) with at least 30X reads, an FDR ≤ 0.05 and with at least a 5% methylation difference. We followed the US National Institutes of Health Roadmap Epigenomics Project Guidelines using 30x sequencing depth in order to achieve a conservative estimate of methylation (Nih Roadmap Epigenomics Mapping Consortium, 2011; Hansen et al., 2012). CompEpitool (Kishore et al., 2015) was used to annotate the differentially methylated CpG sites to the human genome. The bisulfite conversion rate was calculated using methylKit (Akalin et al., 2012). The correlational analysis and clustering analysis were performed using default setting in methylKit for CpG sites in all samples.

### Gene Pathway Enrichment

A list of differentially methylated genes identified by the DMC analysis was further explored using the Kyoto Encyclopedia of Genes and Genomes (KEGG) (Kanehisa and Goto, 2000) to examine functionally annotated gene pathways. The enrichment analysis was performed using the Molecular Signatures Database (msigDB) (Subramanian et al., 2005; Liberzon et al., 2011) with significant enrichment defined by an FDR ≤ 0.05.

## Results

### Quality of the Samples and Reproducibility of the Data

**FIGURE 2:**
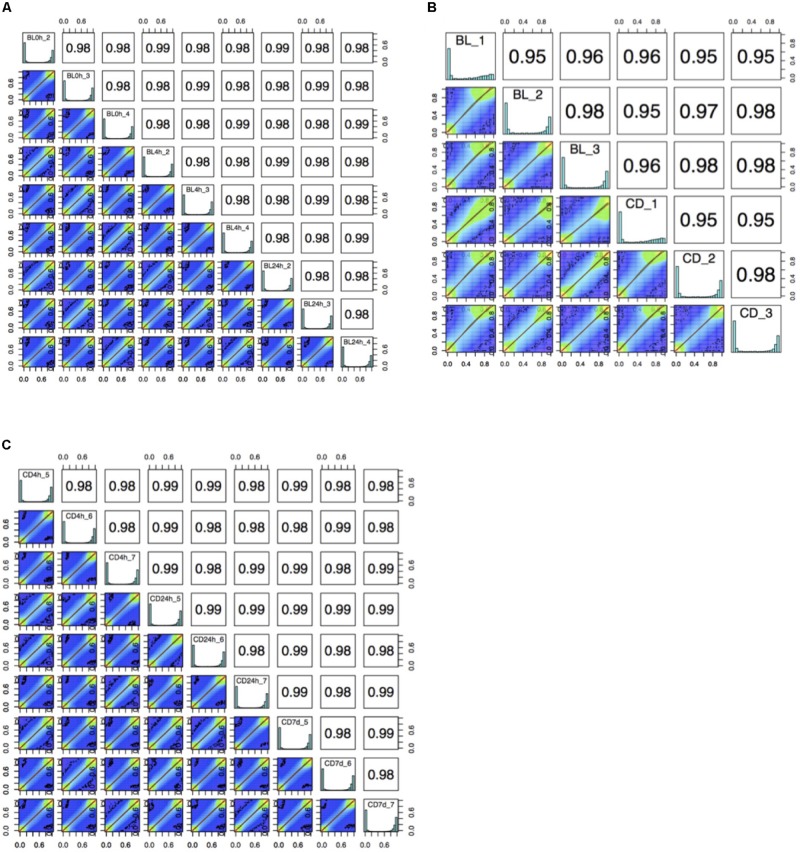
A matrix of correlation coefficients and a set of scatterplots showing the relationship between samples for **(A)** comparisons of whole cord blood for matched samples at 0 h, 4 h, and 24 h (BL, whole cord blood, *n* = 3), **(B)** comparison of whole cord blood and dried blood spots for matched samples (BL, whole cord blood; CD, dried blood spots in cards, *n* = 3) and **(C)** comparisons of dried blood spots for matched samples at 4 h, 24 h, and 7 days (CD, dried blood spots in cards, *n* = 3).

**FIGURE 3:**
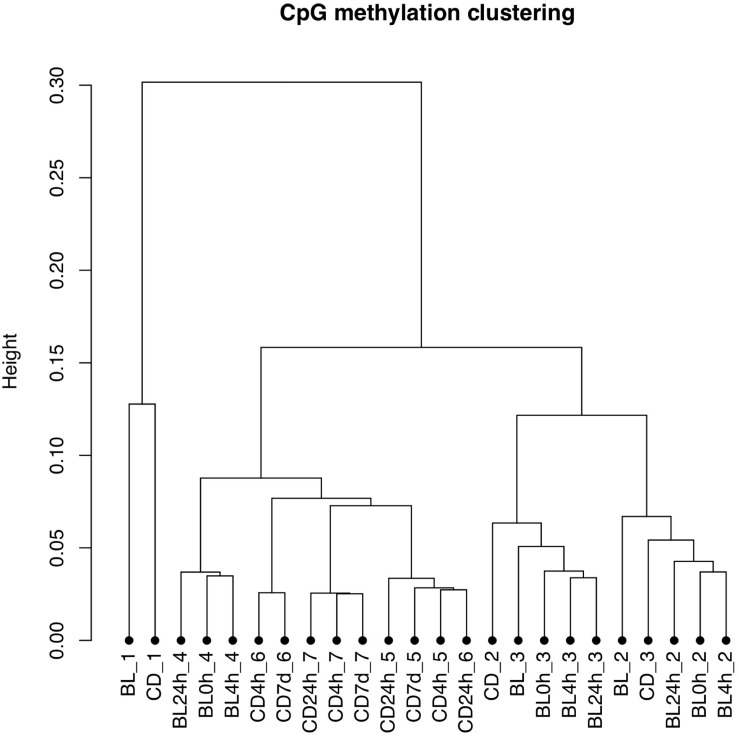
Unsupervised clustering plot based on the methylation values. Samples from the same individual (BL, whole cord blood; CD, dried blood spots in cards) are labeled with the same number. The storage times are indicated 0, 4, and 24 h for timepoints for comparisons of whole cord blood and 4 h, 24 h, and 7 days for timepoints for comparisons of dried blood spots.

The bisulfite conversion rate was greater than 99.4% for all of the samples. DNA methylation profiles were compared between whole cord blood collected at 0, 4, and 24 h (Figure 1A), whole cord blood collected and dried blood spots (Figure 1B) and dried blood spots collected at 4 h, 24 h, and 7 days (Figure 1C) collected from matched individuals. Figure 2 provides a matrix of correlation coefficients for each experiment showing pair-wise comparisons between all samples. We found that the correlation coefficients were greater than 0.8 for all pair-wise comparisons, indicating high levels of reproducibility in our dataset (Bock, 2012; Figure 2). In addition, unsupervised clustering analysis revealed strong similarities across the sample types within each individual. DNA methylation differences between individuals were sufficient to discriminate matched pairs from unrelated samples (Figure 3). These effects were also reflected in higher correlations for sample types from the same individual compared to sample types across different individuals (Figure 2).

### Examination of *ex vivo* Sample Handling on DNA Methylation in Whole Cord Blood

We examined whole cord blood samples left at 4°C for either 4 h or 24 h *ex vivo* (Figure 1A) compared to blood frozen immediately after collection. These comparisons yielded no significant differentially methylated CpG sites meeting our criteria (i.e., FDR ≤ 0.05, ≥5% difference in methylation). Supplementary Table S1 shows the full list of DMCs at FDR < 0.05 for whole cord blood. Complete Blood Count (CBC) analysis revealed that the distribution of different types of nucleated cells were similar across three subjects and within the expected range for neonates, including nucleated red blood cells (nRBCs) that are known to have a unique hypomethylated DNA methylation profile (Supplementary Figure S1; de Goede et al., 2016). Of note, the absolute numbers of nRBCs were 0.2, 0.15, and 0.1 10E9/L, constituting 1.3, 1.3, and 1% of the total blood cell count, respectively, for each subject.

### Comparison of DNA Methylation in Whole Cord Blood and Dried Blood Spots

Next, we examined whole blood frozen immediately compared to blood spots dried for 4 h prior to freezing (Figure 1B). We identified 134 differentially methylated CpG sites that had greater than 5% methylation differences with FDRs < 0.05 (*n* = 3 per condition). Supplementary Table S2 shows the full list of DMCs at FDR < 0.05 for the comparison of whole cord blood with dried blood spots. When annotated, these differentially methylated CpG sites corresponded to 23 genes (Table 1). We performed gene set functional analysis using the Kyoto Encyclopedia of Genes and Genomes (KEGG) pathways to identify common gene pathways associated with this gene set and found no significant gene pathway enrichment, either with the total gene list or gene lists separated by genomic regions (i.e., gene list with differential methylation in promoter regions or gene list with differential methylation in gene-body regions). We identified two genes with multiple CpG sites differentially methylated between whole blood and dried blood spots. TMEM183A (*transmembrane protein 183A*) showed 7 CpG sites, all hypermethylated, with methylation differences of 21.49 to 27.59% in the promoter region. PROZ (*protein Z, vitamin K-dependent plasma glycoprotein*), showed 6 CpG sites, all hypomethylated with methylation differences of −6.16 to −34.16% in the gene body. Supplementary Table S2 provides the full list of differentially methylated CpG sites with associated genic regions and CpG locations.

**TABLE 1:** Differentially methylated CpG sites associated with genes between whole cord blood compared to dried blood spots from the same subjects.

Gene	# of CpGs	Avg. Meth Diff% (Range)	Genic Location
TMEM183A	7	25.2 (21.5 – 27.6)	Promoter
PROZ	6	−24.1 (−6.2 – −34.2)	Genebody
ABL1	2	8.6 (6.5 – 10.7)	Genebody
C11orf53	2	−16.1 (−9.7 – −22.4)	Genebody
CAMK1D	2	−14.8 (−10.1 – −19.5)	Genebody
COX16;SYNJ2BP-COX16	2	−8.3 (−6.2 – −10.3)	Genebody
DMRTA2	2	−4.2 (−14.7 – 6.3)	Genebody
MSLN	2	4.7 (−19.0 – 28.5)	Genebody;promoter
RALBP1	2	−9.7 (−9.6 – −9.8)	Genebody
ZNF101	2	7.5	Promoter
FAM20C	1	−9.4	Genebody
FAM228B;FAM228A	1	−24.0	Genebody;promoter
FASN	1	−6.8	Genebody;promoter
FILIP1	1	16.6	Promoter
GFI1	1	−19.9	Genebody;promoter
GSX1	1	12.5	Promoter
LINC00441	1	5.0	Promoter
MBP	1	5.3	Genebody
PIGQ	1	6.0	Genebody;promoter
RAB6B	1	9.4	Promoter
SAMD11	1	−38.5	Genebody
SIRPA	1	5.3	Genebody
SLC38A10	1	−9.0	Genebody;promoter

*A list of differentially methylated genes is shown sorted by the number of differentially methylated CpG sites.*

### Examination of *ex vivo* Sample Handling on DNA Methylation in Dried Blood Spots

Finally, we examined CpG methylation in blood cards left to dry for 24 h or 7 days at room temperature prior to freezing compared to dried blood spots that were immediately frozen after the 4 h drying phase (Figure 1C). There were no differences in CpG methylation after 24 h of blood card storage at room temperature. We identified 2 differentially methylated CpG sites meeting our criteria after 7 days of storage at room temperature compared to the 4 h sample. Supplementary Table S3 shows the full list of DMCs at FDR < 0.05 for the comparisons of dried blood spots. When these CpG sites were annotated, one of the CpG sites corresponded to a gene: S100Z (*S100 calcium binding protein Z*) with a methylation difference of 6.48% (88.70% at 4 h vs. 82.22% at 7 days). Supplementary Table S4 shows each of the differentially methylated CpG sites along with their associated genic regions and locations.

## Discussion

In this study, we used time-course sampling combined with genome-wide sequencing of gene regulatory elements to examine the influence of handling procedures prior to sample storage on DNA methylation profiles in neonatal blood. We found that different periods of incubation of either whole cord blood or dried blood spots at room temperature had very little effect on DNA methylation profiles. In addition, minimal differences in DNA methylation were detected between whole cord blood immediately frozen compared to blood spots dried for 4 h prior to freezing. DNA methylation profiles in dried blood spots were also similar to those of whole cord blood, except for a small subset of genes. Our findings suggest that DNA methylation profiles are resilient to conditions typical of sample handling procedures for blood collected for research and clinical assessments. These data should provide important information for future prospective and retrospective human studies investigating DNA methylation modifications in whole blood. Importantly, in the present study samples were matched across different time conditions, as they were from the same cord blood samples obtained from the same individuals. The counts for nRBCs were similar across subjects, and these values were similar to mean values previously reported for term babies without complications (Christensen et al., 2011). The results obtained using RRBS are known to correlate strongly with those from commonly used microarray approaches (i.e., illumina450K arrays). Notably, however, the use of RRBS in this study increased by 10-fold the range of genome coverage compared to genome-wide microarray approaches. In addition, we used a threshold of methylation differences >5% in our comparisons (although, we also provide data for the full range of differential methylation meeting an FDR < 0.05). This threshold approximates the detection limits of other platforms such as DNA pyrosequencing (Mikeska et al., 2011). Therefore, we expect that our results will be generalizable across commonly used DNA methylation analysis approaches targeting gene regulatory elements.

Among 23 genes that showed differential methylation between whole cord blood and dried blood spots, we identified only 2 genes with multiple differentially methylated CpG sites; TMEM183A (*transmembrane protein 183A*) and PROZ (*protein Z, vitamin K-dependent plasma glycoprotein*), which showed 7 and 6 significant differentially methylated CpG sites, respectively. TMEM183A is a gene of currently unknown function. The PROZ gene encodes a liver vitamin K-dependent glycoprotein and is synthesized in the liver that is then secreted into the plasma. The protein encoded by PROZ plays a role in regulating blood coagulation by inhibiting activity of coagulation protease. Deficiencies in PROZ protein are associated with an increased risk of ischemic arterial diseases (Lichy et al., 2004, 2006). Our results indicate that the genes that were most affected by air-drying blood were not related to biological processes that are of interest in most studies examining biomarkers of maternal exposures in cord blood.

Our findings contrast with the reported effects of sample handling on gene expression. It has been well documented that sample handling conditions influence the transcription of hundreds of genes (Hartel et al., 2001; Rainen et al., 2002; Baechler et al., 2004; Debey et al., 2004). These genes are involved in the activation of stress and inflammation-induced pathways, cell-cycle progression and apoptosis, which may confound the interpretation of gene expression results obtained from blood. Although DNA methylation is considered a relatively stable epigenetic modification (Bird, 2002), recent studies suggest that DNA methylation modifications at some loci are more dynamic than previously thought. For example, longitudinal analysis has shown that DNA methylation at some sites oscillates across the circadian cycle (Oh et al., 2018). However, we found that DNA methylation profiles are stable and resilient to *ex vivo* handling conditions in whole cord blood as well as in dried blood spots, at least in gene regulatory elements assessed with our approach. Four genes that were previously shown in an analysis of gene expression to be sensitive to *ex vivo* storage (Baechler et al., 2004) also showed differential methylation in dried blood spots compared to whole blood in the present study (Table 1). These genes included FASN (*fatty acid synthase*), GFI1 (*growth factor independent 1 transcription repressor)*, MBP (*myelin basic protein*) and RALBP1 (*ralA binding protein 1*), suggesting that these genes may be sensitive to handling conditions at the level of gene expression and DNA methylation.

Notable strengths of this study are the use of a within-subjects design and a conservative read number (30X), although this study is not without limitations, including small sample size, which may have impacted our ability to detect very small changes in DNA methylation. Also, as sequencing reads obtained using RRBS are enriched at gene regulatory elements with a high CpG content relative to other loci, it may be that larger differences related to sample handling conditions occur outside of these regions. Finally, although the focus of this study was on typical handling conditions associated with the collection of clinical samples, we recognize that future work is needed to study the full range of conceivable handling conditions that may affect DNA methylome profiles. The current study focused on procedures for sample handling in clinical settings, which typically range from immediate to overnight sample storage time (up to 7 days in the case of blood cards) followed by freezing. A limited number of other studies have examined archival samples of dried blood. For example, Hollegaard et al. (2013) showed in matched samples of adult whole blood from two individuals, that methylation signatures were highly correlated between the whole blood and dried blood spots stored at room temperature for 3 years. Also, Joo et al. (2013) showed that matched samples of 5 adult buffy coat and dried blood spots stored at room temperature were highly correlated after 3 years of storage at room temperature. Although methylation analysis is feasible in older archival samples [e.g., samples collected >20 years ago (Hollegaard et al., 2013)], the ability to match these archival samples with immediately collected samples is a limitation in our current understanding of the potential impact of a wider range of storage conditions on DNA methylation. Nevertheless, with the recent advent of high-throughput epigenomic technologies that require high quality DNA, our findings on the relative stability of DNA methylation with varying sample handling and preparation conditions – including those pertaining to blood cards – can help guide current and future studies. There is emerging appreciation of the utility of cord blood and dried blood spots collected at birth as a resource for mechanistic, prognostic and diagnostic epigenetic studies of the Developmental Origins of Health and Disease, and our findings support such a strategy.

In summary, we have demonstrated that DNA methylation profiles are quite stable in whole cord blood, as well as, in dried blood spots following various *ex vivo* manipulations. Our findings stand to increase the scope of biological resources suitable for epigenome-wide association studies. This further adds to the potential use of neonatal dried blood spot samples in screening beyond genetic assessments and paves the way for population-based studies of epigenetic modifications after birth.

## Data Availability Statement

The data discussed in this article have been deposited in NCBI’s Gene Expression Omnibus (Edgar et al., 2002) and are accessible through GEO Series accession number GSE146468 (https://www.ncbi.nlm.nih.gov/geo/query/acc.cgi?acc=GSE146468).

## Ethics Statement

The studies involving human participants were reviewed and approved by the Mount Sinai Hospital Research Ethics Board. The patients/participants provided their written informed consent to participate in this study.

## Author Contributions

AS and SM designed the research and wrote the manuscript. AS and BK performed the research. AS analyzed the data. KM provided the materials and supervised the patient enrolment and acquisition of biological samples. SM supervised the research. All authors approved the final version of the manuscript.

## Conflict of Interest

The authors declare that the research was conducted in the absence of any commercial or financial relationships that could be construed as a potential conflict of interest.

## Footnotes

1 https://www.bioinformatics.babraham.ac.uk/projects/trim_galore/

2 https://github.com/nugentechnologies/NuMetRRBS/blob/master/trimRRBSdiversityAdaptCustomers.py
