# Supplementary figures and images for: Impact of ex vivo Sample Handling on DNA Methylation Profiles in Human Cord Blood and Neonatal Dried Blood Spots
Source: Front Genet. 2020 Mar 24;11:224. doi: 10.3389/fgene.2020.00224 (PMC7106936; doi:10.3389/fgene.2020.00224)

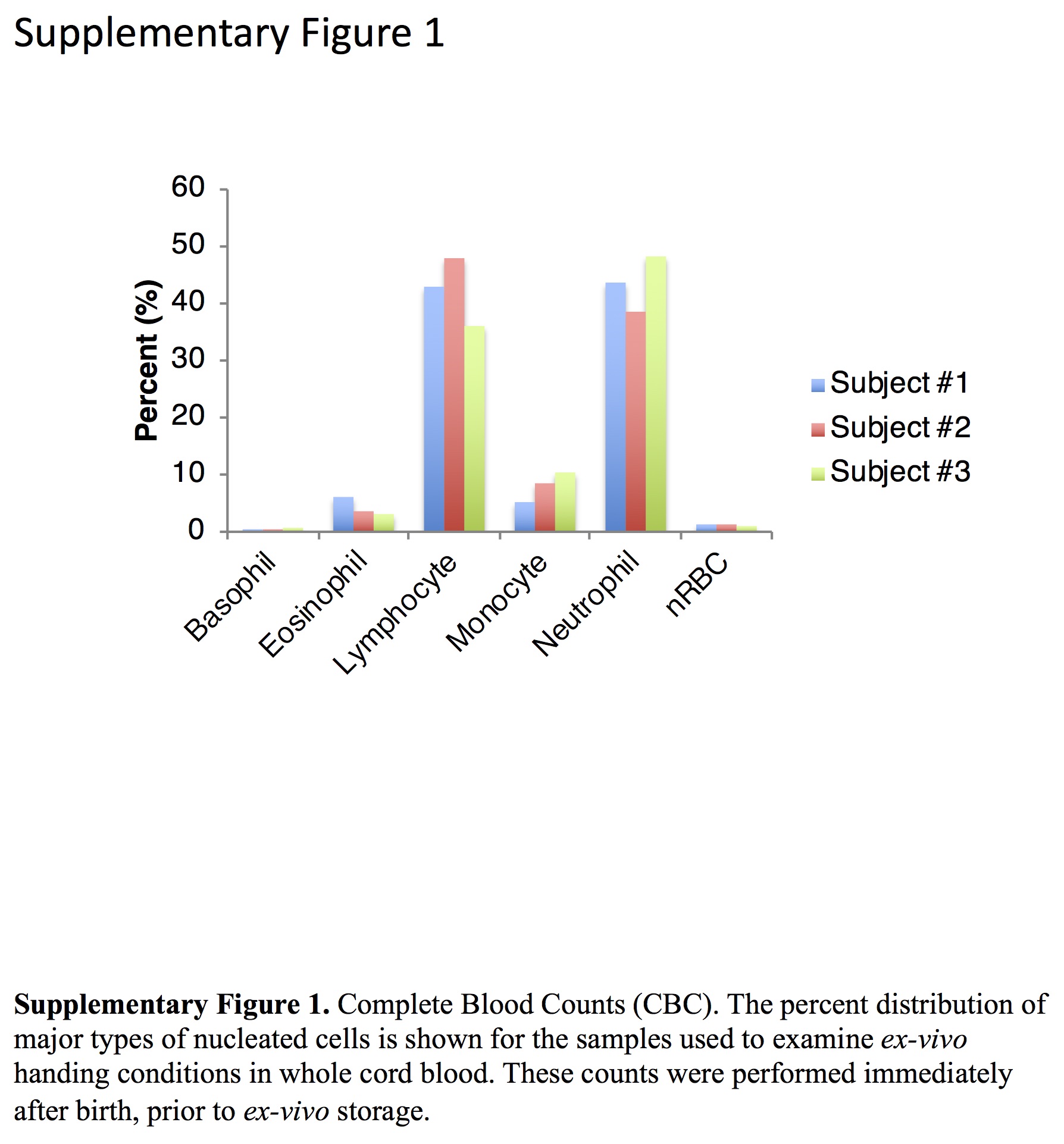

Supplement: Supplementary file 1 [file Image_1.JPEG]
